# Changes in the Activity of the Erector Spinae and Gluteus Medius Muscles with the Presence of Simulated Lower Limb Dysmetria

**DOI:** 10.3390/s24041223

**Published:** 2024-02-14

**Authors:** María Benito de Pedro, Ana Isabel Benito de Pedro, Ángela Aguilera Rubio, Jose Luis Maté Muñoz, Juan Hernández Lougedo

**Affiliations:** 1 Department of Physiotherapy, Faculty of Health HM, Camilo José Cela University, 28692 Madrid, Spain; mbenito@ucjc.edu (M.B.d.P.); angela.aguilera@ucjc.edu (Á.A.R.); jlougedo@ucjc.edu (J.H.L.); 2 Department of Radiology, Rehabiilitation and Physiotherapy, Complutense University of Madrid, 28040 Madrid, Spain; jmate03@ucm.es

**Keywords:** leg length discrepancy, low back pain, gluteus medius, erector spinae, CMJ, electromyography

## Abstract

(1) Background: Leg length discrepancy (LLD), regardless of its origin, is a very common pathology that can contribute to low back pain. Various authors have pointed out its relationship with the lack of activation of both the gluteus medius (GM) and the ipsilateral erector spinae (ES). The purpose of this study was to identify the activation of the ES and GM with different simulated LLDs, correlating this activation with LBP. In turn, we evaluated whether ES and GM activity has an effect on jumping ability using a CMJ test. (2) Method: A sample of healthy subjects was selected to whom an artificial LLD was applied using 0.5, 1, and 1.5 cm insoles. These three heights were measured using EMG while the subjects walked and performed a counter movement jump (CMJ). The measurements of the insole heights were carried out in random order using a Latin square. Muscle activation patterns were recorded for 30 s at each of the insole heights while the patients walked at 5.7 km/h and they were compared with the maximum voluntary contraction (MVC), both on the ipsilateral and contralateral sides. These muscles were then measured under the same circumstances during the performance of the CMJ. (3) Results: We found statistically significant differences in the flight heights in both the CMJ and DJ. In the comparison, significant differences were found in the flight heights of the CMJ and the DJ using the 5 mm insoles, and in the case of the DJ, also without insoles, with respect to the MVC. We found statistically significant differences in the activation of the GM with the differences in insoles, but not in the activation of the Es in relation to the different insole heights. (4) Conclusions: Insoles of different heights caused activation differences in the medius on the side where the insoles were placed. We can relate this difference in activation to LBP. In relation to the ES, no significant differences were found in the activation of the ipsilateral side of the insole.

## 1. Introduction

Leg length discrepancy (LLD) has been a controversial topic among researchers and clinicians for many years [1].

LLD is divided into anatomical and functional types. Anatomical LLD, defined as a structural deformity that originates from true bony differences in the lengths of the legs [2], is the anatomical difference between the lengths of the two extremities from the head femoral to the distal tibia; this can be congenital or acquired [2]. The most common congenital causes of LLD are Silver Russell syndrome, proximal femoral deficiency, congenital neoplastic diseases, syndromes associated with vascular malformations (Klippel–Trénaunay–Weber syndrome), and some overgrowth syndromes, like Proteus syndrome. There are also acquired causes of LLD, such as trauma, tumor, radiation, and infection, or paralytic disorders, like polio [3,4].

Functional LLD is defined as an asymmetric alteration in leg length, which is not necessarily the result of a real difference in bone length and can be caused by an alteration in the biomechanics of the lower extremities due to weakness, muscle shortening, or a static or dynamic misalignment of the mechanical axis [5]. Functional LLD can occur anywhere from the superior aspect of the ilium to the inferior aspect of the foot and is usually the result of pelvic obliquity related to adaptive soft tissue shortening, joint or muscle contractures, ligament laxity, or axial malalignment [1]. As the pelvis rotates, the legs are straightened in apparently different [1].

LLD, regardless of its origin, can cause significant misalignment of the lower extremities [5].

Asymmetries in gait kinematics in patients with LLD have been associated with different degrees of true LLD [6]. Essentially, a pelvic drop and hip adduction are evident in the static stance phase [7]. The pelvis drops and rotates in the sagittal and/or frontal planes [8], and the long leg usually compensates with the pronation of the foot, adduction, and flexion of the hip and knee [6]. These changes in kinematics contribute to pelvic tilting toward the side of the short leg in the frontal and sagittal planes, increasing its load [9]. 

In their study, Azizan et al. showed greater loading forces in the shorter limb during walking (4).

Common compensatory mechanisms of the shorter leg include supination or plantarflexion of the foot, along with hip extension and knee hyperextension [10] The compensatory mechanisms of the longer leg include foot pronation and knee flexion [11], which lead to the aforementioned pelvic tilt in the coronal and sagittal planes, postural abnormalities (inclined shoulder), and a hyperextended knee in the shorter leg and flexed knee in the longer leg [10].

All of these mechanisms are understood as compensatory strategies in both the shortest and longest limbs (5).

These asymmetries are often accompanied by neuromuscular adaptations, as Murrel and Triano reflect in their studies [3,12].

Various authors have focused on neuromuscular adaptation for long-term dysmetria; the standing balance of subjects with LLD was evaluated from their dysmetria [3].

Several studies have shown that anatomical LLD is very common, occurring in up to 70% of the population, and they have also pointed out that discrepancies of >2 cm affect approximately 1 in every 1000 people [5]. In their 2005 systematic review, Knutson et al. assessed the prevalence of LLD using radiographs to perform measurements, and they revealed that 90% of the population has differences greater than 1 mm in the bone lengths of their. Approximately 50% of the study population had a maximum discrepancy of 4 mm in length between their limbs, and only 20% were affected by a variation of >9 mm [13]. Certain authors have stated that an LLD of up to 25 mm does not have a detrimental effect on function [14,15].

In 2018, a study by Khamis et al. showed that even mild simulated LLDs cause changes in the kinematics of the lower extremities, thus suggesting that LLDs of 5–10 mm should not be ignored [16]. Betsch et al. [17] observed that simulated LLDs of less than 15 mm caused pelvic tilt and torsion; however, no significant changes in the spinal position were detected. Young et al. [18] revealed that artificial LLDs greater than 15 mm caused lateral flexion of the trunk (evoking functional scoliosis). Asymmetry of more than 20 mm resulted in a significant increase in pelvic obliquity and torsion, as well as a clear increase in the superficial rotation and lateral deviation of the spine. 

Unequal loading on the shorter limb was studied by Walsh et al. [2] showing a pelvic obliquity toward the shorter side, which is the most common compensatory mechanism.

To compensate for pelvic obliquity, Cummings et al. observed that lumbar scoliosis occurs with the convexity directed toward the shorter limb [2].

Several publications affirm that LLD is a significant factor in various pathological and physiological conditions, including a higher incidence of stress fractures and injuries in the lower extremities of athletes [19], knee osteoarthritis [20] and abnormal biomechanics of the foot [21]. It has also been proven that LLD affects the function of certain muscle groups of the lower limbs [5,22]. In their investigation, Abate et al. showed greater activity in the muscles of both lower limbs [23]: the tibialis anterior, gastrocnemius, quadriceps femoris, and latissimus dorsi. Other authors have evidenced in their publications an amplified activity of the lower extremity muscles and intrinsic lumbar back muscles in an electromyography during gait [24,25]. 

Different authors associate LLD with low back pain (LBP) [26,27] and sciatica [28], functional scoliosis in children [29], and back injuries among runners [30]. In addition, degenerative changes in the spine have been described, and a reduced intervertebral disc height was associated with an increased loading of the zygapophyseal joints [31]. 

In a controlled study using superficial electromyography (sEMG), Decarreaux and colleagues found that the stabilizing muscles of the trunk (rectus abdominis, multifidus, ES, and external oblique) took longer to reach their maximum strengths during an isometric flexion and extension exercise in people with chronic low back pain compared to healthy individuals [32]. An explanation of this, in line with other similar studies, is that their findings were due to muscle fatigue [33].

In their 2023 publication, Murat et al. concluded that the erector spinae (ES) had a greater contribution to lumbar extension compared to the multifidus. Therefore, its contribution to low back pain would also be greater [34]. 

LBP is a common complaint among adults worldwide, with an estimated lifetime incidence of 60–80% in the adult population [35]. Many theories have been developed about the etiology of LBP. In addition to direct damage to certain anatomical structures, intervertebral discs, facet joints, ligaments, nerves, and muscles, several authors believe that the contribution of the biomechanics of the lower extremities in the development of LBP is the key to understanding this pathology [35].

The gluteus medius (GM) has also been implicated in the development of LBP [36]. The GM is one of the main pelvic muscles that actively participates in the control of motion in the frontal and transverse plane and hip [37], improving the stability of the lumbopelvic–hip complex [38].

Reduced GM muscle strength creates a propensity in individuals to exhibit LBP [39]. This alteration in GM strength and its relationship with LBP was also addressed by Kanchanomai et al. in 2015 [40]. 

One of the most used methods to quantify muscle activation and neuromuscular fatigue through mechanical variables is to calculate the loss of muscle capacity to generate force after performing a countermovement jump (CMJ) [41] Muscular fatigue, as measured through the CMJ test, which involves muscle stretching–shortening cycles, reflects a reduction in muscle–tendon stiffness induced by structural damage to the tendon insertions, impairing CMJ performance [42,43].

In 2022, Carroll et al. associated the presence of active or latent GM trigger points with hip weakness in adults with chronic nonspecific LBP [44].

Recently, Fraçz et al. studied the cross-sectional area of the GM, confirming existing atrophy on the same side of the pain in patients with LBP [45]. 

Physiotherapeutic treatment is considered relevant in LLD to control the symptoms and understand the importance of activating the GM in order to maintain pelvic balance with therapeutic exercise [46,47]. Treatment includes stretching, since pelvic obliquity creates shortening due to the adaptation of the soft tissues, the contracture of joints or muscles, or the laxity of ligaments [48].

LLDs smaller than 20 mm are usually asymptomatic and represent a normal variant. For these people, internal shoe lifts are added with a thickness corresponding to the discrepancy, which normally ranges between 5 mm and 15 mm. External heel lifts are typically more comfortable for patients with LLDs of 15 to 20 mm [29].

We are not aware of any study on how induced artificial LLDs could change muscle activity in the ES or GM.

The aim of the present study was to investigate whether modifying the lower limb length with different foot insoles of 0.5, 1, and 1.5 cm in a normal population has an effect on ES and GM activity, and as a consequence, on LBP. The secondary objective was to evaluate whether ES and GM activity has an effect on jumping ability, as assessed through CMJ.

## 2. Materials and Methods

### 2.1. Study Design

In this cross-over trial, we evaluated the muscle activity of the ES and GM muscles using superficial electromyography. This activity was recorded while the participants walked and performed a CMJ. This study was approved by the Ethics Committee of Clinical Research of Camilo José Cela University and conducted in accordance with the Declaration of Helsinki. All subjects provided written informed consent.

### 2.2. Participants

This study was carried out at Facultad de Ciencias de la Salud de la UCJC at the physiotherapy center in the city of Villanueva de la Cañada (Madrid). The study protocol was supervised and approved by the Institutional Ethics Committee of Clinical Research of Camilo José Cela University and conducted in accordance with the Declaration of Helsinki. All subjects gave written informed consent.

After identifying a series of inclusion criteria, 34 participants ultimately joined the study. The inclusion criteria were as follows: no history of lower extremity injury and no history of disabling LBP that might have affected their walking for 6 weeks prior to testing [35]. 

The volunteers had no problems in the range of motion of the joints of their lower limbs [35] and they were screened for a series of exclusion criteria: pelvic obliquity due to a functional leg length discrepancy, and obesity with a body mass index (BMI) of 35 > kg/m^2^ [17]. They were screened for limb length discrepancy using a standard flexible tape measure, with each limb measured from the medial malleolus to the anterior inferior iliac spine, as recommended by Beattie et al. [49].

The participants were randomly assigned to 1 of 3 groups using random assignments based on a computer-generated randomization routine using EpiData software version 3.02 (EpiData Association, Odense, Denmark), and the allocations were concealed with sealed, numbered, tamperproof, and opaque envelopes, which were opened only after the participants consented to participate.

A “Latin square design” was used. In the first group, the participants first used insoles of 5 mm. In the second and third test sessions, the participants in this group used insoles with thicknesses of 10 and 15 mm, respectively. In the second group, the participants used insoles with thicknesses of 10, 15, and 5 mm in the first, second, and third test sessions, respectively. In the third group, the participants used insoles with thicknesses of 15, 5, and 10 mm in the first, second, and third test sessions, respectively.

**Figure 1:**
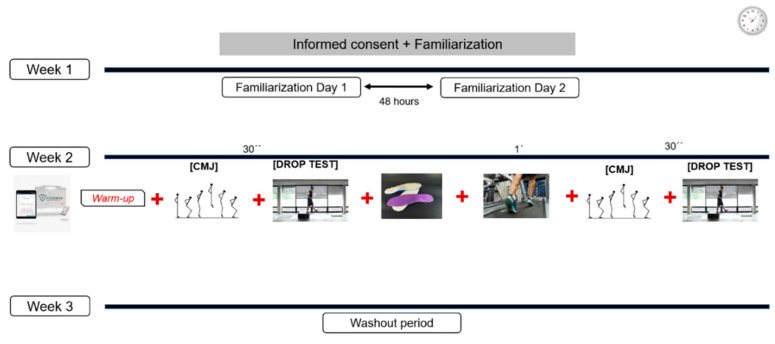
Study design.

Between the test sessions, there was a washout period of at least 1 week to allow the muscles to relax. Figure 1 depicts the study design.

### 2.3. Sample Size Calculation

Version 3.1.9.2 of G*Power© statistical software (from Dusseldorf University, Düsseldorf, Germany) was used to calculate the sample size using the Mann–Whitney U test for the means of the 2 samples according to the non-parametric data distribution. In order to achieve large effect size differences between the groups, an effect size with a Cohen’s d of 0.70 was used. In addition, an error probability of 0.05 for α, a confidence interval (CI) of 95%, a β error probability of 20% with a power analysis of 80% (1-β error) for two-tailed hypothesis, and an allocation ratio for N2/N1 of 1/1 were used. Therefore, a total sample size of 25 participants was calculated, and according to a possible 25% loss to follow-up, a total sample size of 20 participants was required. 

### 2.4. Instrumentation and Data Collection

#### 2.4.1. EMG Measurement

EMG was recorded during a gait cycle using 3 types of insole (0.5, 1, and 1.5 cm). Two muscles in the lower back were investigated bilaterally: ES and GM. 

For measurement, we used a superficial EMG mDurance R system (mDurance Solutions SL, Granada, Spain), which is a portable sEMG system that consists of three parts [50]: (1) A Shimmer3 EMG unit (Realtime Technologies Ltd., Dublin, Ireland), which is a bipolar sEMG sensor for the acquisition of muscle activity. Each Shimmer sensor is composed of two sEMG channels, with a sampling rate of 1024 Hz. The Shimmer unit applied a bandwidth of 8.4 kHz, and the EMG signal resolution was 24 bits, with an overall amplification of 100–10,000 V/V. The electrodes used were pre-gelled Ag/AgCl with a diameter of 10 mm [50]. (2) The mDurance (Android) mobile application, which receives data from the Shimmer unit and sends it to a cloud service [50]. (3) The mDurance cloud service, where the sEMG signals were stored, filtered, and analyzed, generating the reports [50].

**Figure 2:**
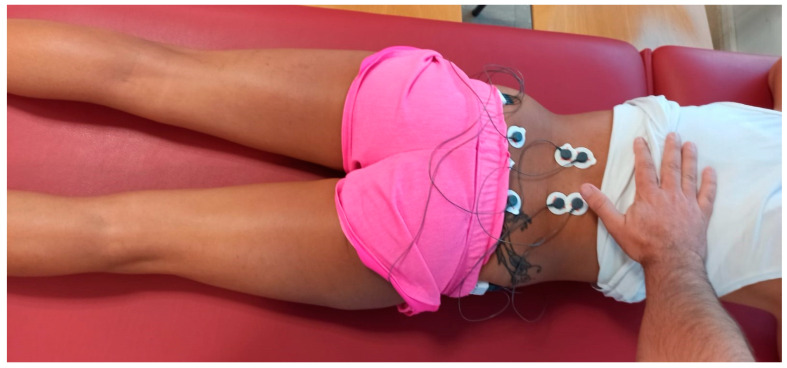
Placement of electrodes for ES (SENIAM).

**Figure 3:**
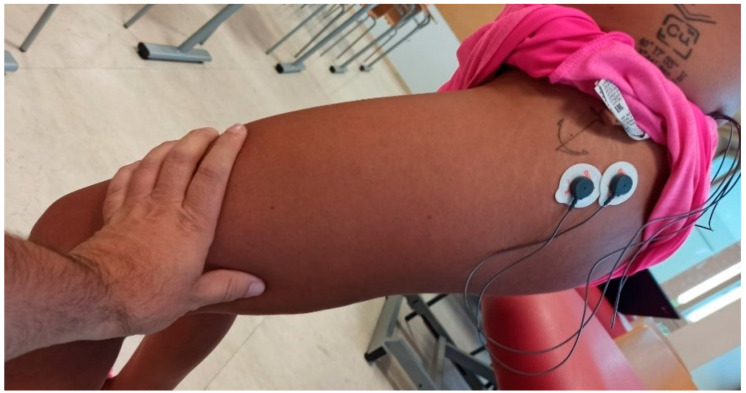
Placement of electrodes for GM (SENIAM).

The surface electrode placement was carried out following the Surface EMG Recommendations for the Non-Invasive Assessment of Muscles (SENIAM) [51], bilaterally and parallel to the muscle fiber direction. Figure 2 and Figure 3 depict the placement of the electrodes.

The electrodes were secured with hypoallergenic adhesive plaster [51] to prevent their movement.

All shoes used by an individual subject were of the same make, model, and size [24]. Shoe lifts with heights of 0.5, 1, and 1.5 cm and constructed from crepe material were placed in the midsole of the right shoe. 

##### MVC Measurement

First, the participants were asked to perform a voluntary isometric maximum contraction (MVC) [52] after receiving precise instructions on how to perform the MVC of the muscle in question. Three maximal attempts of 6 s each were performed, separated by 3 min to recover from the MVCs of the ES and GM. The best performance was chosen for the statistical analysis [53].

The investigator asked the participants to perform a maximal contraction of the GM by abducting their hips while he applied manual pressure to stabilize hip movement with one hand and the ankle with the other [54].

For the ES, one of the techniques used to perform an MVC [55] involves extending the trunk while lying prone. This technique was not utilized because it was noted in preliminary testing that this was quite uncomfortable for the participants, and the signal output was significantly affected by fatigue, even with up to 3 min of rest between contractions. Instead, a reference voluntary contraction was used. The modified Schöber (MMS) skin distraction technique [56] uses a flexible tape measure to measure the amount of skin distraction over the lumbar spine. The participants were verbally encouraged while performing the evaluation. The purpose of this test is to allow the researcher to compare the maximum amplitudes with submaximal tasks, such as walking and performing CMJ [57].

##### EMG Recording during Walking

The participants first walked on the treadmill to become used to it before the test. Subsequently, the subjects walked continuously for 3 min with each insole height on a motorized treadmill at a speed of 5.17 km/h [58].

The activity patterns of the muscles were recorded for each condition for 30 s, with five minutes of recovery between the experiments, during which each subject remained seated [59].

##### CMJ and Drop Test

Muscle activation and fatigue were assessed in the legs by measuring the vertical reaction forces (0–10 kN; sampling velocity 0.5 kHz) in a CMJ [60] performed on a portable 92 × 92 × 12.5 cm force platform (Quattro Jump model 9290AD; Kistler Instruments, Winterthur, Switzerland) before the test and post-test. After the warm-up and before starting the test, the participants stood on the platform with their legs extended and with their hands on their hips. For the jump, the legs were first flexed to 90° (eccentric action) and then explosively extended in a coordinated manner (concentric action), aiming for maximum height. During the flight stage, the knees were extended. Contact with the ground was made with the toes first. During the test, the subjects were instructed to keep their hands on their hips and to avoid sideways displacements. After finishing the test, the participants were encouraged to keep moving in the cool-down phase while they waited to perform the CMJ tests. For the drop test, the participants had to perform a jump from a height of 30 cm with unipodal support, performing it with their dominant leg and then with their non-dominant leg.

### 2.5. Statistical Analysis 

The Shapiro–Wilk test was used to analyze the normality of the variables. A one-way ANOVA with repeated measures was performed, contrasting it with Mauchley’s test of sphericity. In the case of rejecting the sphericity hypothesis, the univariate F statistic was used, adjusting it with the Greenhouse–Geisser corrective index. In the case of finding significant differences among the measurements, the Bonferroni post hoc test was applied. Furthermore, to find differences in the electromyographic activation using different insoles between the right and left side, a two-factor ANOVA (side and insoles) was performed with repeated measures for the insoles factor, applying Levene’s statistic (Levene’s test) to assess the homogeneity of the variances. The effect of the insoles × side interaction was also analyzed by applying the Bonferroni post hoc index for pairwise comparison. All data were expressed as the mean (M), standard deviation (SD), and confidence intervals (CI). In addition, the statistical power (SP) of the data and the effect size, known as partial eta squared (ηp2), were determined, categorizing the magnitude of the difference as trivial (≤0.01), small (0.01 ≤ a < 0.06), moderate (0.06 ≤ a < 0.14), or large (≥0.14). The level of statistical significance was *p* < 0.05. SPSS Statistical Package version 25.0 (SPSS, Chicago, III) was used. 

## 3. Results

After performing the jump tests, significant differences were obtained in the heights of flight of the CMJ [F(4.76) = 4.686, *p* < 0.05)], and the heights of flight of the DJ performed with the left leg [F(4.76) = 4.262, *p* < 0.05)], with no differences found in the power of the CMJ nor in the heights of flight of the DJ performed with the right leg (*p* > 0.05). Table 1 shows the detailed results.

When analyzing the electromyographic activation in percentages (Table 2), significant differences were only found in the GM using the different insoles [F(2,132) = 4.262, *p* < 0.05)], without significant differences found between the sides [F(1,66) = 0.149, *p* > 0.05)] nor in the insoles × side interaction [F(2,132) = 0.140, *p* > 0.05)]. The analysis carried out for the ES was along the same lines as that carried out for the GM, with no significant differences found between the thicknesses of the insoles or between the right and left limbs.

Significant differences were found between the electromyographic activation of the GM using the different insoles [F(3,114) = 62.394, *p* < 0.05)], without significant differences being found between the sides [F(3,114) = 0.010, *p* > 0.05) ] nor in the insoles × side interaction [F(3,114) = 0.008, *p* > 0.05)]. When analyzing the pairwise comparison, no significant differences were documented for the electromyographic activation performed among the insoles. For the electromyography performed on the ES, no significant differences were observed between the activation of the sides [F(3,114) = 0.541, *p* > 0.05)] nor in the insoles × side interaction [F(3,114) = 0.569, *p* > 0.05)]. When using the Bonferroni post hoc test to perform the pairwise comparison, no significant differences were observed in the activation among the insoles (*p* > 0.05).

## 4. Discussion

Based on our findings, there were no significant differences observed in the activation of the ES muscle when comparing the contralateral side and different types of insoles, as indicated by Frieberg’s research. The study conducted by Frieberg revealed a strong association of symptoms in the lumbar area and hip with the leg length discrepancy, with chronic pain or recurrent sciatica mainly occurring on the longer leg side in the majority of their sample population (79–89%) [7].

Our results do not correlate with those of Vink and Huson, in contrast to another study that examined the impact of an asymmetrical insole height on treadmill walking, where an increased activity time was observed in the intrinsic lumbar muscles during the heel strikes of the artificially raised limb. Our study did not yield similar results. 

In their 2015 study, Murray and Azari [61] observed a correlation between discrepancies in leg length and L5-S1 degenerative joint disease in both sexes.

In the shorter leg, the downward obliquity of the pelvis increases the lateral flexion of the lumbar spine and rotation to the opposite side [62]. The combination of these postures or adaptive movements to LLD causes great structural stress on the soft tissues of the column [63]. In their 2005 article, Defrin et al. described the importance of the orthopedic correction of LLD in helping to improve chronic LBP [27]. 

Kakushima et al. discovered that individuals with artificial leg length discrepancy exhibited an asymmetrical movement in lumbar lateral flexion during walking, specifically toward the side of the shorter leg. This compensatory mechanism was utilized to address the LLD and was not observed in their study on normal gait. Consequently, this increased stress from lateral flexion may contribute to degenerative spinal disorders [64].

More recent observational studies of asymptomatic subjects found an association between fatigue of the postural trunk muscles and low back pain [65]. It is important to highlight that the exhaustion of these muscles was identified as occurring before the onset of pain, indicating that it was not a result of the pain. These findings do not align with our own research.

Vink and Karssemeijer [66] examined the muscle activity of the lumbar region in a healthy group during walking, specifically looking at the simultaneous activation of both sides of the lumbar muscles when the heel makes contact with the ground. It is hypothesized that maintaining a symmetrical activation pattern within the lower back muscles when walking is necessary to balance the forces exerted on the torso. Prolonged periods of asymmetric patterns may be considered dysfunctional and can potentially lead to pain in the lumbar area [67]. 

Bird et al. conducted a study using sEMG measurement and found that the activation of the ES muscle occurred earlier when bilateral 20 mm heel pads were used [35]. In a study conducted by Knutson and Owens, individuals with leg length discrepancy were found to have significantly shorter endurance times for the ES muscles compared to those without LLD. However, it should be noted that different insole heights were not used as a comparison in this particular study (Biering–Sorensen test) [13]. 

In subjects with LPB, two studies found less muscle activity in their GM [68,69] another study found more activity in those with LBP [36], and studies have even been published where no differences were shown in the GM muscle activity between subjects with and without LBP [70,71,72] According to one study, individuals with low back pain demonstrated reduced GM muscle activity [72].

In 2010, Nelson Wong et al. found earlier activation of the GM compared to other trunk muscles in subjects with LBP [65].

The present study aimed to investigate the impact of different insoles on biomechanics, specifically muscle activity, during CMJ and drop landing tests. By considering the individual potential and characteristics of each participant, the researchers aimed to determine if the examined mechanisms aligned with their power, force, and anthropometric traits. One key finding of this study was that the height of the drop landing did not necessarily correspond to the peak height reached during the CMJ. 

This discrepancy suggests that there are differences in the ground reaction forces and muscle activity between these two types of movements [42,43,73]. In the drop test, we found differences between the ipsilateral and contralateral sides with respect to the insoles, higher flight heights being obtained on the contralateral side of the insole with respect to the ipsilateral side, produced by the muscular adaptations to counteract the difference in height generated with the insoles [43,73].

Nevertheless, despite observing minor improvements in jump height during the CMJ and drop tests, there were no significant variations observed between the basal condition and when using a 5 mm insole. Despite an increase in muscle activation, it is evident that this alone is insufficient to generate an elevation in jump height induced by the GM [42,73,74].

## 5. Conclusions

The aim of this research was to examine the impact of using foot insoles with varying heights (0.5, 1, and 1.5 cm) on lumbar RS and GM activity in individuals without any existing back pain. This study aimed to determine whether these modifications in lower limb length would have any subsequent influence on the occurrence of low back pain.

Varying the heights of the insoles led to variations in the activation levels of the GM on the side where the insole was placed. This disparity in activation could be linked to low back pain. On the other hand, there were no notable discrepancies observed concerning ES activation between the same side as the insole and its contralateral counterpart. As a result, we cannot establish a connection between ES activity and LBP occurrence.

Based on the examination of the secondary objective, it is evident that an enhanced activation of GM muscles with a 0.5 cm insole leads to a higher jump height compared to the initial state. However, there were no noticeable disparities in the jump heights when comparing the different insoles.

## 6. Limitation

One significant constraint of our study is that the participants in our sample did not exhibit actual dysmetria, but rather a simulated form of it. Consequently, we were unable to validate the discrepancies in muscle activation reported in various articles concerning real instances of dysmetria.

## Figures and Tables

**Table 1:** Analysis of CMJ and DJ using different insoles in the twenty subjects analyzed.

Variable	Without Insole (M ± SD, 95% IC)	5 mm Insole (M ± SD, 95% IC)	10 mm Insole (M ± SD, 95% IC)	15 mm Insole (M ± SD, 95% IC)	η_p_^2^	PS	*p* Time
Height CMJ (cm)	27.75 ± 4.80 (25.50–29.99)	29.20 ± 5.22 * (26.76–31.64)	29.24 ± 6.17 (26.35–32.13)	29.27 ± 6.63 (26.17–32.73)	0.198	0.848	0.007
Poser CMJ (watts·kg^−1^)	840.90 ± 289.09 (705.60–979.19)	821.79 ± 129.77 (761.05–882.52)	821.06 ± 136.18 (757.03–885.09)	821.67 ± 152.54 (750.27–893.06)	0.013	0.078	0.660
Height DJ left (cm)	11.55 ± 3.81 ‡ (9.76–13.33)	11.63 ± 4.07 ‡ (9.73–13.54)	12.31 ± 4.77 (10.07–14.54)	12.80 ± 3.81 (11.01–14.58)	0.183	0.812	0.011
Height DJ right (cm)	11.53 ± 3.25 (10.01–13.05)	12.21 ± 4.04 (10.32–14.12)	12.79 ± 2.79 (11.48–14.10)	12.86 ± 3.55 (11.19–14.52)	0.101	0.485	0.114

CMJ—counter movement jump; DJ—drop jump; * significant differences with pre-exercise values (*p* < 0.05); ‡ significant differences with maximum voluntary contraction (*p* < 0.05). M ± SD—mean ± standard deviation, CI—confidence interval.

**Table 2:** Analysis of electromyographic activation expressed as a percentage with respect to MVC of GM and ES muscles using different insoles.

Variable	Limbs	5 mm Insole (M ± SD, 95% IC)	10 mm Insole (M ± SD, 95% IC)	15 mm Insole (M ± SD, 95% IC)	*p* Time η_p_^2^ SP	*p* Group η_p_^2^ SP	*p* Time × Group η_p_^2^ SP
Gluteus medius (mV)	Left	35.46 ± 43.11 (17.67–53.24)	30.36 ± 30.31 (17.89–42.83)	28.97 ± 26.98 (16.47–41.47)	0.027 * 0.059 0.643	0.690 0.002 0.068	0.814 0.002 0.069
	Right	38.80 ± 59.47 (21.02–56.59)	33.67 ± 41.64 (21.20–46.14)	34.25 ± 44.00 (21.76–46.75)			
Erector spinae (%)	Left	39.21 ± 40.20 (27.47–50.95)	40.50 ± 48.96 (25.68–55.32)	35.74 ± 47.62 (21.04–50.43)	0.987 <0.001 0.052	0.354 0.013 0.151	0.706 0.005 0.105
	Right	30.35 ± 27.13 (18.61–42.10)	29.87 ± 36.70 (15.05–44.68)	33.04 ± 37.63 (18.34–47.73)			

* Significant differences with the rest of the values on the left side and right side (*p* < 0.05); M ± SD—mean ± standard deviation; IC—confidence interval; SP—statistical power; η_P_^2^—partial eta squared.

## Data Availability

Raw data will be made available upon request to the corresponding author.
